# Congestion of the Brain in Cholera

**Published:** 1854-11

**Authors:** James M. Newman

**Affiliations:** Health Physician


ART. II.—*Congestion of the Brain in Cholera.* By JAMES M. NEWMAN, M. D., Health Physician. Read before the Buffalo Medical Association, October 3d, 1854.

Cholera has been the prolific source of an innumerable host of reports, essays, and theories; and so limitless is their number that we may well imagine, if it were possible to fathom the depths of the mysteries in which this terror-inspiring epidemic is enshrouded, we should have long since exhausted it of all interest, stripped it of its terrors, disarmed it of its power, and rendered the narrative of its visitation dull as a thrice told tale. But, as much as we have learned of this pestilence, we unfortunately know too little yet, to deprive the subject of interest, or to make it an unprofitable source of study.

Until the time shall arrive when, either the plague shall cease to visit us, or we shall be conscious of our ability to follow out all of the windings of the labyrinthian mazes which envelop the whole history of its origin, cause, mode of seizure, and treatment, it becomes the duty rather of all those who have the opportunity of studying this disease to add to the sum of previous labors, great as that is, with the hope that from the labor of some one, or from an analysis of the aggregate, there may be evolved a spark which may be kindled into a light sufficient to illume the whole subject and reveal the deepest depths of the now almost impenetrable darkness.

Impelled by such considerations, I propose to add to the garnered stores, the results of my observations made during the recent prevalence of the disease in this city, including such reflections as the study of the cases have suggested; with the course and results of treatment, and proposing such indications of diagnosis and treatment as a careful consideration of these cases would seem to indicate.

I propose, however, to confine my observations to that complication of the disease depending upon some morbid condition of the brain, manifesting itself in symptoms of congestion, and known under the various names of congestion of the brain, congestive fever, consecutive fever of cholera, typhus, or typhoid fever in the course of cholera, &c.

It is, perhaps, proper to add that these observations have been made mainly at the bedside of patients coming under my notice and charge as a Health Officer; and that they have been treated at the Hospital of the Sisters of Charity; and that, as far as nursing is concerned, they have had not only the advantage of skillful and experienced attendants, but have received all the kind care and attentive watching that the Sisters could possibly bestow, affording as favorable a field for the observation of this disease as could be well found. At the same time it should not be forgotten, that the patients were principally drawn from our foreign population, many of them emigrants just landed in our city, and that their peculiar habits, circumstances, and idiosyncrasies, came in as so many elements to disturb and complicate the disease.

The general features of the disease are, however, undisturbed, and will, I believe, upon comparison, be found to bear a close resemblance to the cases occurring in any part of the city, or under any condition of life; and I fancy any peculiarities manifested here in the epidemic under consideration, will be recognizable in any other locality of its visitation. Reports all bear testimony to its extreme malignancy, and to the impotency of the best directed efforts put forth for the relief of the unfortunate victims of the pestilence.

There is a class of cases in which there never occurs the pallid, sunken countenance generally seen in cholera, and so characteristic of the disease. If reäction is established, the bright, if not ruddy complexion of the patients seems to belie the assertion that but a few hours before they were at the very mouth of the grave.

This redness of the face is attended by several peculiarities. In some cases it presents the appearance of a bright blush diffusing itself over the whole cheek. In others it is a deeper red, and not so largely diffused; the color toward the center of the patch becoming more and more intense, a bright circumscribed spot is manifest upon a field of less intensity. The small cutaneous vessels are minutely injected, and the general appearance is not unlike that caused by the immoderate use of alcoholic stimulants, or frequent and long exposure to the sun.

This redness of the skin, depending upon the injection of the vessels, is very persistent. It remains during the whole period of treatment, and long after convalescence. It seems as if it required to be removed by some process of reparation.

The eyes are more or less injected. The intensity of this injection is very various, and is increased with the persistency of the case, and its tendency to a fatal termination. The conjunctivæ in some cases are injected in their lower halves only, that portion covered by the upper lids remaining singularly clear.

If convalescence be not speedily established, the vomiting and purging continue, or, if for a time checked return again. Drinks, medicines, and nourishments, are all alike rejected by the stomach; and the matters thrown up cease to be a mere colorless liquid, but are more or less tinged, generally being yellow, sometimes greenish.

The discharges from the bowels are frequent, and assume in a greater or less degree the consistency, odor and appearance of ordinary diarrhœa. The discharges sometimes are very offensive. This condition is not always observable, the evacuations occasionally continuing throughout the disease rice-water. But I believe this is more generally observable in cases of a rapid termination, and in cases where a relapse has been sudden from a condition of comparative convalescence, and partakes of the character of a second attack of cholera.

As the vomiting and diarrhœa continue, the strength wanes; the color of the face deepens, or extends in surface; the eyes are more and more injected; the patient becomes drowsy and inclined to sleep; the stupor gradually increases, and finally, profound coma becomes established, terminating in death, or recovery after long treatment and a tedious convalescence.

The condition just described is Congestion of the Brain in the course of Cholera.

The flushed face, and injected eye, are the premonitory symptoms of, and the indications pointing out the tendency of the disease to such a termination, and warn us against any treatment which may precipitately hurry it on, and conduce to hasten our patients to the grave.

I would solicit for the consideration of these conditions of the face and eye, more than a casual notice. I have come to regard them as valuable diagnostic signs; and as they occur early in the disease, look upon them as affording us an unerring index to our course of treatment. With these marks upon our patients we have Congestion of the Brain, in some form or other, to contend with. And fortunate, indeed, shall we be if our patient prove to be lightly affected, or our medication wards off the impending evil.

I have already spoken of the persistency of the vomiting and purging attendant upon this condition of the patient, and as the concomitants of the flushed face and injected eye. Upon these morbid manifestations I propose to theorize some.

At this stage of the disease the vomiting and purging cease to be choleraic only in their character and indications. The vomiting here I believe to be the result of the irritation of the brain, the effects of its congested state only, or of a state of inflammation coëxisting with the congestion,—a precise pathological point I am unable to settle; a question to be, perhaps, only settled when all other kindred questions, as to the definitive boundaries and domains of irritation and inflammation are drawn and meted out.

The diarrhœa depends partly upon the same morbid impressions, and partly upon the exudation of the blood's serum through the coats of the intestines, in whose membranes some lesion is effected by the onset of cholera, either by some imperceptible change in its vital action, or in absolute lesion of structure.

The vomiting is more readily, speedily, and effectually controlled than the diarrhœa.

If my view of the pathological conditions of this stage of cholera be correct, we have arrived at once at a very important stage of the discussion as to the indications of treatment for this condition of our patients.

I fear there has been not a little error committed, from regarding this condition as differing in no particular from cholera, or as indicating no modified treatment, except as considering it merely a very intractable case, requiring a marshaling of our forces in greater array and strength.

As I have come to regard this *stage* of cholera, as it perhaps may be properly called, the complication of the disease has assumed an importance paramount to the original disease. We are here to cease to regard and to treat the disease as cholera merely; but we are to merge this consideration into a knowledge of the fact that we have a brain affection to treat, sufficiently serious to destroy our patient. We shall then cease to medicate the disease as having a local habitation in the stomach: a point of attack which I apprehend it is the too prevalent practice to direct our aim; only too often, I fear, to waste our time and resources.

If this view be correct, the indications of treatment are very apparent. The vomiting and attendant symptoms, if controlled, are to be managed by our attacks upon the brain, and not upon the stomach. In other words, we have converted it from a stomach disease to a brain affection. Our medication is consequently accordingly influenced.

Opium and its compounds cease to be safe remedies, and must be altogether abandoned. Calomel becomes an important article of our materia medica. Shaving the head and the application of cold; local bleeding by cups or leeches, all are indicated. Stimulants and tonics are to be given gradually and watchfully. We must support and strengthen our patient without goading the brain, and pumping into its already repleted, sluggish channels, a still larger supply of blood.

The symptoms of coma in some slight cases, or in the onset of the attack, yield to cold water, or to the ice cap. Cupping and leeching are required in others, and afford relief. Blisters I have used; but they are slow in their action, and valuable time is lost; and besides, they irritate the patient and annoy him with the pain of the subsequent sore, so that latterly I have not so frequently employed them.

At this point I will introduce the notes of several cases, a number sufficient to illustrate the character and course of the stage of cholera under consideration, and to exhibit the severity of its seizure, the variety of its complications, and its resistance to treatment. Reserving such farther reflections as the observations of this complication may suggest, for notice in another place.

Case I.—A German woman was received in the Hospital, sick with cholera. The disease was so far controlled that collapse never became complete, and partial reäction and restoration occurred. But the vomiting and diarrhœa continued. The vomiting was intractable for several days, the stomach rejecting medicines, food, and drinks. She was observed to become drowsy, which increased from day to day with the vomiting, until it became so complete that she merely gazed vacantly around when shaken and loudly spoken to. Her face was flushed at this stage. Its condition at the onset is not now remembered. Her pulse was about 100.

At this stage of the case her head was shaved, and the ice cap applied, and five drops of the solution of the iodo-hydrg. potass. given every three hours. This treatment was commenced in the morning, and at evening the vomiting was controlled, and it never again returned. The coma became less profound, and cleared perceptibly from day to day, until she became completely conscious, and in the full exercise of her faculties. Her stomach retained food and medicines.

Her condition constantly improved under a tonic and dietic course of treatment for a week, when, upon an evening visit to the ward, I found her pulse had gone up since morning to 120, her skin was cold, and the powers of life rapidly failing. She died during the night, all disturbance of the intellectual faculties being absent, and she sank away as in a slumber.

Case II.—August 10th, a family of German emigrants, consisting of five persons, arrived in this city, and were attacked with cholera. The cases of three may serve to illustrate our article.

They arrived in the morning in apparent good health, and during the afternoon the father was attacked, and died before the next morning. He had no particular medication: some medicine having been obtained from a drug store and administered.

During the same night the wife was attacked, and, upon being called in the morning, I found her completely collapsed in one bed, and the corpse of her husband in another.

I gave her calomel, grs. xx., morph., gr. i., and had her removed to the Hospital.

Reäction never ensued. She became comatose, and, notwithstanding every measure was adopted to relieve her, she died in the course of the next day. Her condition too plainly indicating the state of the brain.

On the 14th of August, I was called to see two daughters of the above, young women. They had removed into another portion of the city. They had every appearance of having always enjoyed robust health.

They were both attacked during the night, or early in the morning. At the time of visit, about 10½ o'clock, A. M., though both were vomiting frequently, and the diarrhœa was severe, the older one seemed to be most severely attacked by the disease, and to be the most completely in its power. There was that appearance of prostration, with the blue congested state of the lips and face indicating how fearfully had already been the inroads of the disease.

I gave each calomel, grs. xv., morph. gr. i., and directed their removal to the Hospital. The medicine had no influence in either case in checking the discharges. The hopeless condition of the elder sister was very soon apparent. From a state of listlessness she became drowsy, then comatose. The stupor increased in intensity until the next day, when she died. (No opiates were given to either after their admission to the Hospital.)

A *post-mortem* examination revealed the congested, turgid state of the bloodvessels of the brain, and an effusion of several ounces of bloody serum at the base of the brain, and within the canal of the medulla oblongata.

For the want of a better term, I designate this case and that of the mother, on account of the suddenness and intensity of the coma, and the amount of lesion present, as apoplectic. The amount of effusion and degree of lesion too plainly point out the utter hopelessness of being able, by any treatment, to relieve our patients in such seizures.

The younger sister, for many hours after her admission, seemed to fail. The vomiting and diarrhœa continued. She was becoming gradually more and more drowsy. Still there was an apparent greater degree of vitality remaining, as manifested by her bright, clear complexion, which was lighted up by a flush extending over the whole cheek. This bright complexion is but too often the mask which conceals the deadliest foe. We prognosticated no other result than the sorrowful fate of the other members of her family. Her pulse, at this time, was 120, small and feeble.

Our medication was more particularly directed to the head. Opiates were withheld, and, with one single exception to be mentioned hereafter, not a particle was given during the whole time of her treatment. Her head was early shaved, the ice cap kept continually on, and leeches repeatedly applied to the temples. The first application of leeches was made during a night when the intensity of the coma was so great as to threaten death before morning, and the flow of blood was followed by a perceptible amelioration of these threatening symptoms. Each subsequent application manifested a corresponding improvement. The vomiting ceased with the amelioration of the head symptoms, though the discharges from the bowels continued until convalescence was fully established.

In the mean time calomel, one and a-half grains, quinine, two grains, and tannin, three grains, were given every three hours for forty-eight hours, extending over three days. On the third day, the discharges from the bowels became dysenteric, owing, as I supposed, to the effects of the calomel. At this point the calomel was immediately suspended.

The symptoms which, since the commencement of the calomel and the leeching, had been gradually lightening up, became very manifestly improved upon the commencement of the bloody discharges, and convalescence went on gradually from this time, but continuously. The bowels were controlled by astringents, given both by the mouth and by enemas.

The pulse was the last to acknowledge the gradual restoration of the patient. It was on the onset 120, and it continued this number for many days after all the other symptoms pointed toward recovery. Finally, three or four beats a day gave away, until I had the pleasure of finding it fallen to 100, and, on the third day thereafter, (Aug. 28th,) the still greater pleasure of finding it down to 80, from which time she required no farther care.

To illustrate how easily the condition of the brain under consideration may be affected by opiates, the following circumstance may serve as an example:

The attendant one day becoming somewhat anxious at the presence and persistence of the bloody discharges, gave a starch enema with a drachm of laudanum. It had been scarce thrown up the rectum before the patient manifested its influence. She fell into a profound slumber, very much to the alarm of her kind attendant. It produced, however, no injurious effects. This was the only opiate administered in any form during her continuance in the Hospital.

Case III.—Aug. 15th, Patrick, an Irishman, employed upon the lake, was attacked; he obtained some medicines, one powder, of a respectable physician.

On the 16th he came regularly under the care of another gentleman, a judicious practitioner, who continued to attend him until the morning of the 18th, when he was conveyed to the Hospital.

He had been collapsed, but had rallied, reäction seemed complete, and he was regarded as a fair subject to prognosticate a recovery. His diarrhœa and vomiting still continued when he entered the Hospital, but there was an absence of that appearance of great prostration apparent in cholera patients generally. His intelligence was perfect; he answered questions correctly; was calm, and apprehensive of no danger.

He had been in the ward some little time before my visit, and had been under the eye of the observant Sister having the ward in charge. Upon my examining him she remarked that he was now under the influence of some medicine, and she believed he was strongly influenced by a large dose of some opiate. His complexion was florid and destitute of the pallor of cholera; and the conjunctivæ of both eyes strongly injected, and the cornea bright.

Adopting the hypothesis of these appearances being the effects of opium, none was given, nor was a particle of opium, or any of its compounds, given during his treatment.

Brandy, quinine, tannin, and animal broths, were used during the day. The next day the vomiting still continued, and the fluids ejected were deeply yellow, and the evacuations from the bowels assumed more of the appearance of common diarrhœa. The tendency to sleep was increasing, and the face was becoming more and more florid, with the deeper, dusky red spot of the cheek.

His head was shaved, and the ice cap applied, and calomel, in one and a half grain doses, was added to his other medicines, and administered every two hours.

At evening his vomiting was checked, and he seemed better; a state of apparent improvement which continued some ten or twelve hours, when he relapsed, becoming, finally, completely comatose. The redness of the face also increased, extending down upon the neck, and attained a depth of color between that of rubeola and scarlatina. His chest manifested, in a less degree of intensity, the same color. The coma became more and more profound, until the powers of life failed, and he died on the fourth day after his entrance, and on the seventh from his first attack. His pulse, a few hours before death, was scarce 90.

His former medical attendant saw him frequently during his hospital treatment, and stated that he was deterred upon his first visit, in consequence of the injected condition of the eyes, from giving him any opiates, and that he gave him none whatever during his attendance.

A *post-mortem* examination revealed an effusion of several ounces of bloody serum, about the base of the brain, and medulla oblongata. The vessels upon the surface of the brain, were injected of a deep, dark color. There was very little fluid in the ventricles, and the substance of the brain was injected but slightly more than natural. The blood was extremely fluid, and flowed freely from every part of the body. The organs of the chest and abdomen exhibited nothing abnormal, except a close and firm adhesion of the pericardium to the heart.

Case IV.—Aug. 24th. A German, aged 31 years, a brickmaker by trade, toward evening was brought into the Hospital in a most hopeless state of collapse, and in a condition in which speedy death seemed almost certain.

He had indulged in a spree the day before, and, although having a diarrhœa in consequence, he commenced work the next morning, and continued to labor until noon, when he was compelled to yield to the symptoms of cholera, which at this time were developed in their fullest severity.

When admitted into the Hospital his vomiting and diarrhœa were very severe, the discharges being almost incessant. Morphine, calomel, and diffusable stimulants, were administered. The stomach rejected these, as every thing else, as soon as placed upon the tongue. Morphine, to the amount of three grains, and calomel, fifteen grains, were given, before any seemed to be retained, or the vomiting and diarrhœa were controlled. Sinapisms were also applied.

The symptoms finally yielded, the stomach and bowels became quiet, and warmth diffused itself over the surface.

25th. Upon my visit this morning, I found reäction fully established, the stomach and bowels were controlled, the pulse good, the general appearance favorable, and the patient expressed himself as feeling very well. There was no stupor, and the condition of the intellectual faculties was as clear and active as they ever were in a man not naturally very brilliant.

The only cause for anxiety in the case, that I could detect, was the *bright, florid* condition of the face; a condition I pointed out, and expressed a fear that it was a state which might yet be the source of much difficulty.

He was directed through the day, quinine in two grain doses every three hours; brandy as occasion required; and animal broths; and a watch to be kept for head troubles. Opium, in all its forms, was to be withheld.

26th, 11 o'clock, A. M. The patient spent yesterday comfortably, and rested well during the night. But this morning he seems not so well, and for the last three or four hours has been quite uneasy.

The skin is hot and dry, the pulse is 115, he is restless, has some pain of stomach, and there are feelings of general *mal aise.* The color of the face is deeper, there is a slight headache; but little or no redness of the conjunctivæ. His mental faculties are yet unimpaired.

7 o'clock, P. M. My patient is better and expresses himself much relieved. His skin is cool and moist, the pulse has fallen to 96, and the general feeling of pain and sickness has passed off.

To day he has taken spts. nitr. eth. ʒj, once an hour, and quinine, grs. ix., during the day. His head has been shaved, and ice water kept constantly applied. His florid complexion has not yet entirely left him.

27th. Feels very comfortable. Skin warm but moist, with a tendency to free perspiration. Pulse 90.

28th. The pulse is this morning down to 80, and convalescence seems fairly established. The tonic and supporting plan of the first day is still continued. Opiates in no form have been given since the first night of treatment. The flushed face still continues.

29th. Is begging this morning for his clothes, that he may dress and be up about the wards. Says he is so tired of the bed that he cannot sleep at night, and grumbles grievously because they are withheld for another day.

30th. Is up and dressed, and sitting out upon the open verandah in the rear of the Hospital.

He left a few days afterward, entirely recovered. An increase of color, still, was evident.

Case V.—Aug. 28th. Michael Kelley, an Irish boy of about 17 years, was conveyed to the Hospital this morning about 8 o'clock, by his mother, in a condition of complete collapse.

Yesterday he indulged in green apples and cakes, and shortly after went in swimming. Toward the close of the afternoon, diarrhœa set in, followed soon by vomiting, both of which have continued during the night, and when received into the Hospital, he was completely collapsed. The discharges still continued, and his appearance indicated only a speedy dissolution.

His face *is flushed, eyes injected,* and has slight headache. He has received no medication until since his entrance into the Hospital. His father died last week with cholera, and the week previous a sister-in-law died of the same disease.

29th. Reäction slowly commenced yesterday, and this morning he is much better. The vomiting is checked, but the diarrhœa still continues. The surface is warm, moist, and the pulse is full. He still complains of great thirst, has some headache, the flush of face is yet present, and eyes continue injected. He took yesterday morphine, gr. ½, calomel, grs. x., with brandy, ice, beef essence, and had counter-irritation over stomach and bowels.

30th. Toward evening of yesterday and during the night, he was drowsy and inclined to sleep. Cold was immediately applied to his head, and is still continued.

This morning he seems quite comfortable. His diarrhœa has entirely ceased, nor does he vomit. He still continues very thirsty. His face is still flushed, but the eyes are less injected. He has some headache. Pulse 100.

The ice cap was directed to his head. He is taking no opiates.

31st. This boy's appearance is very good, and his pulse is but 80. Still he has a return of the vomiting and diarrhœa. His own willfulness, perhaps, has much to do with it. He complains much of thirst, and commits the greatest indiscretions, in trying to obtain more drink than is allowed him. In the absence of the nurse this morning, he got up and went out upon the verandah.

℞ Calomel and quinine, $\overline{aa}$ gr. i.

Every three hours, put dry upon the tongue.

Sept. 1st. Vomiting and diarrhœa are entirely checked this morning, and general condition improved. The injection of the eye has subsided, but the face yet retains its bright red flush. His pulse is 80, but is irregularly intermittent. Heat of skin not more than natural. He still complains of thirst.

3d. Has gone on well since last entry, and convalescence seems fairly established.

His recovery was perfect.

CASE VI.—Sept. 2d. Charles O. H., an Irish boy, aged 7 years, was conveyed to the Hospital this morning from a deep, damp, dirty cellar, in the lower part of the city. He was attacked yesterday with symptoms of cholera, and has received no treatment, until his reception into the Hospital.

He has now copious diarrhœa and frequent vomiting. His eye is deeply sunken, skin cold, and pulse small, and general appearance is not very promising. There is a light blush diffused over the whole countenance, and the eyes are deeply injected.

I directed his head to be shaved, and ice water applied, with sinapisms to the abdomen, and brandy as he could bear it.

3d. The vomiting this morning is entirely checked, and the diarrhœa controlled. His general appearance is far more promising than it was yesterday. His eyes are much less injected.

4th. Improving. No diarrhœa or vomiting. He has not had a particle of medicine of any kind.

5th. Is this morning inclined to be very drowsy, sleeping continuously. He can, however, be aroused so as to take food and drinks. Directed leeches to the temples, and the administration of quinine and brandy.

6th. His condition is not very materially changed since yesterday. He sleeps continuously, and his eyes are more injected. His pulse is 96. The leeches could not be made to bite yesterday, and a small blister to each temple has been applied.

7th. Did not rally, and died this noon.

Case VII.—A sister younger than Charles, came in at the same time with him, sick with the same disease, and apparently a more unpromising case. She soon became drowsy and comatose, but these symptoms, after some three or four days' continuance, were controlled and relieved by the constant application of ice. Her head was laid upon the bladder of ice as upon a pillow, and her unusual quietude kept it continuously in that position. She recovered. The remedies were principally beef essence and brandy. She never had an opiate.

*Remarks.*—I confess to having been disappointed in the fatal termination of the case of Charles, after the two days of marked and promising improvement. The case, however, as well as that of his sister, serves to illustrate the nondependance of this condition of the brain upon the medication of cholera.

Case VIII.—Sept. 8th, 1854. T., a Bohemian girl, aged 13 years, was admitted into the Hospital in the afternoon. The day previous her mother had died of cholera, and, on the morning of the 8th, an elder sister was attacked with the same disease, and was already in a state of collapse, in which condition she was removed to the Hospital, and died in the course of the next day.

The subject of this record was attacked that morning with diarrhœa, but was still up at the time of removal of her sister, and walked to the Hospital, a short distance from her residence.

She was immediately put in bed, and medicines administered.

9th. Notwithstanding the free administration of calomel and morphine, and the most attentive nursing, she has continued to vomit freely, and have copious evacuations from the bowels during the night, and this morning she is collapsed. Her skin is cold, pulse small, and fingers shriveled. This condition is aggravated by the rain of to-day, and a damp, chilly atmosphere. The patient is extremely restless.

Directed cold to the head, the use of slaked lime about the body and extremities, and the administration of stimulants and tonics, with enemas.

10th. The vomiting is checked, and the diarrhœa is much less. But she is becoming very drowsy, the face is flushed, and the eyes are deeply injected.

The ice cap to be kept continuously applied to the head. Medicines as before.

11th. Is quite comatose. Directed leeches to temples, and the continuance of the ice cap, and

℞ Calomel,

Sulph. quinine, $\overline{aa}$ grs. xij.

Tannin, grs. xx.

Ft. pulv. vi., one to be given every three hours.

12th. Six leeches were applied yesterday to the temples, but one only could be induced to bite, and bled but slightly. Cups were then thoroughly used upon the temples and neck, but no blood could be obtained from the scarifications.

She continued to fail during the day, and died about midnight.

*Remarks.*—The difficulty of obtaining blood by local appliances, is worthy of notice, as one of the conditions attending this form of the disease.

While this patient was conscious, she expressed herself as being much relieved by the ice cap, and that its application was accompanied by the most agreeable sensations. Her extreme and uncontrollable restlessness prevented, in a great measure, its constant application.

Case IX.—Sept. 8th. Jacob Barnard, a young man, a German, was brought to the Hospital sick. He has been sick for several days: nothing beyond this is known, either as to severity of attack, or treatment.

He has evidently, at this time, incipient congestion of the brain. Has no vomiting, but little diarrhœa; skin is dry, and of moderate temperature; strength is yet good, pulse about 80. His face is flushed and eyes congested. He is listless and drowsy, requiring an effort to arouse him.

Directed his head to be shaved, and ice applied.

9th. As there seems to be very little letting up of this drowsy condition, directed his temples and neck to be cupped. This operation was thoroughly performed, but very little blood was, however, obtained. Pulse 72.

10th. Seems more conscious to-day. Has recognized his friends. Pulse 72.

11th. Is more drowsy again, and is restless. Has an increase of diarrhœa. Pulse 72. Prescribed cupping again, and the following:

℞ Calomel, grs. x.
Quinine, grs. xij.
Tannin, ʒss.

Ft. pulv. vi. Give one every three hours.

12th. Is more easily roused to-day than yesterday. His diarrhœa is less, and upon the whole seems slightly improved. He was thoroughly scarified yesterday, but no blood could be obtained. Pulse 72.

13th. His pulse to-day is 88. He can be roused, but his attention can be fixed but for a moment. A blister has been applied this morning to each temple.

The same medicines to be continued. The calomel has not yet made any impression upon his gums.

14th. Can be more easily roused to-day. His diarrhœa seems entirely checked, and has been for near twenty-four hours. His gums are to-day touched with the calomel. The blisters to the temples filled well. His pulse to-day is 96.

Suspend the calomel, but continue the other medicines.

15th. Seems better. Countenance is much brighter than yesterday, and he answers questions readily. Bowels still under control. Pulse 88.

16th. Is wakeful and conscious, but is very weak. Seems more prostrate than at any former period. Pulse 100.

17th. His symptoms and appearance are all much improved. Pulse 100.

18th. There is an increased heat of skin to-day, with an unusual redness of flesh, particularly of the hands, arms and face. Has some diarrhœa again. Pulse 100.

℞ Tinct. kino for diarrhœa.

19th. Improving. Pulse 96. Diarrhœa continues. Cannot take tinct. kino.

℞ Acet. plumb., grs. ij., every two hours.

Has an abscess forming over angle of the jaw on the right side.

℞ Ungunt. iodid. comp., q. s.
Sulph. Morph., gr. j. M.

The above to constitute one application, and to be rubbed into the tumor, and to be repeated two or three times during the day.

20th. Is much better to-day; diarrhœa is checked; pulse 85; tumor of the face is much softer and not so painful.

22d. Is much improved. His pulse is down to 80. The tumor of the face has quite subsided, and without any suppuration, under the use of the ointment.

25th. Continues to improve; sits up. Pulse 80.

28th. Has continued to improve, and is now completely convalescent, except the want of his usual strength. Has walked about the yard for the past two days, and to-day left the Hospital for his own home.

CASE X.—In this, a fatal case, I will introduce the *post-mortem* appearances only. The patient was a German woman, who had lingered for some time, and in whom the coma had been complete for several days. She had recovered from this state of stupor, her stomach retained food and medicines, and she had but little diarrhœa. She notwithstanding continued to fail, and died.

An *autopsy* revealed the following appearances: The vessels of the brain, externally and internally, were turgid and dark colored. Two or three small patches of coagulable lymph were observable on the dura mater, and the membranes themselves seemed slightly thickened. Serum was effused at the base of the brain, and ran freely from the spinal canal. Serum was also largely effused within the ventricles. Upon cutting into the substance of the brain, an infinite number of small bloody points followed the track of the scalpel, and from each one of which a minute drop of bright-colored blood was exuded, so as to stand distinctly above the cut surface of the brain. The blood in all the vessels was extremely fluid, flowing from every divided one with all the freedom of life, and, as seen through the coats of the veins, seemed divided into two distinct portions, the colored being separated from the serous.

In connection with the *post-mortem* appearances of the blood, the following may not be unworthy of notice: Upon the floor a quantity of blood had run down from the table, covering a spot about a foot square. It was observed after a few minutes, that this blood had, by exposure to the air, undergone a marked change in its color. It had, from the dark appearance which it possessed when it first flowed from the severed vessels, become of a bright scarlet, or arterial hue.

An examination of the stomach and intestines, and other viscera, yielded no noteworthy appearances.

I regard the term of typhus, or typhoid fever, applied by the books and in common parlance, to this condition of cholera, as a misnomer. In all the cases coming under my observation, there were none of the symptoms of typhus or typhoid fever present, unless it be the red, dry tongue.

There is no continuous heat of the skin, or regular accessions of fever. If the skin is hot at the beginning of this state, it is but transitory; apparently reäction in excess from the state of previous complete prostration, or collapse of the patient. This soon subsides, or is easily controlled by a diaphoretic. During the most continuous cases under my observation, there was never any heat of surface deserving the appellation of fever. The skin was cool, and if any tendency existed it was to sink below the natural standard.

No petechiæ were in any case present.

There is no delirium. The degree of unconsciousness is of all grades, from simple drowsiness to complete coma. In the first state the patient can be aroused to answer questions; in the severer states, the stupor is complete. The only manifestations of delirium is extreme restlessness, with a disposition to get out of bed.

Neither is there any subsultus tendinum, or picking at the bed clothes.

The severity of the thirst grows less with the lapse of time and progress of the disease.

I cannot but regard this misapplication of terms of far more importance than first appears upon its face. Our treatment, of course, is based upon the supposition of the correctness of our diagnosis, and it must occur upon a moment's reflection, that if the conditions we are considering be not typhus or typhoid fever, then a modification of treatment is indicated and demanded.

These fevers admit of the free use of opiates, and their administration is not only sanctioned by abundant authority, but their employment indicated by the wants of the patient.

In the Congestion of Brain of Cholera, opiates are contraïndicated and forbidden. We have not a condition of the brain simply sympathetic in its character, but the symptoms are those developed by morbid actions, whose seat is within the brain itself. Its vessels are turgid; serum and blood are, perhaps, poured out within its ventricles, or at its base; and so rapid is this effusion, so great the lesions, and extreme the shock occasioned thereby in some of the cases, that their termination is sudden and apoplectic. In others the morbid impressions are more gradual, the powers of life are less rudely attacked, and the vital energies more slowly succomb to the evil; or, perhaps, rally, and convalescence is slowly, but effectually, established.

In these sudden apoplectic cases, effusion, I presume, would always be found, upon examination, to be present, and sufficiently accounts for the fearfully rapid sinking of the patient into the arms of death.

In these more gradual cases how much depends upon irritation simply, and how much upon inflammation, or where, or when, or how we are to draw the line of distinction, must be settled by future investigation.

It can lead to no serious errors in practice to regard the congestion and consequent irritation, as precursors of inflammation with attendant effusion.

I think there may be found in this complication of cholera, as presented in the epidemic of this year, or at least in that part of it which has come under my observation, a key to some of the contrarieties of practice, and a solution to the success or non-success of the same course of practice, in the hands of different practitioners, or when applied to different localities.

I am led to believe that the disease manifests itself differently in different seasons; or it is influenced by some local cause, so that this determination of he disease to the brain is not uniform, but that it may prevail in one year , or in a certain locality, and that another season and a different locality may be exempt from this complication.

At least this is the first year I have met with it. In the epidemic of 1849, in a field of not very limited observation afforded me as an attaché of the Board of Health, among the poor of that part of the city known as the Hydraulics, I did not meet with it. Amid all of the unfavorable circumstances attendant upon the treatment of the disease, often amid the most squallid poverty, and concentrated filth, I did not meet with a single case of congestion of the brain in the course of cholera. Reäction once established was convalescence, and convalescence was perfect restoration in a few days.

In 1852, in a field limited to private practice, I had no congestion of the brain, and recovery was by the same progressive steps as in 1849.

This year I was soon taught that there was some remarkable peculiarities, either in my patients or in the character of the disease I was called upon to treat. I soon learned that convalescence was not a certain guarantee of recovery; nor that reäction once complete was any surety against a subsequent collapse. Discharges checked for twenty-four or thirty-six hours, would frequently break forth again, and reduce them to the verge of collapse. Some, from a state of comparative comfort, and with prospects of speedy recovery, would become drowsy, then comatose, and die; or pass through a long period of treatment and tedious recovery.

To the question so often asked me, Whether the manner of treatment, or the opiates did not tend to produce this state? I can say, without hesitation, No. It was a condition entirely independent of the medication; a condition to be made much worse by improper treatment, undoubtedly. Care was, from the commencement, exercised to avoid *producing* this condition, and as the tendency of the disease to *assume* this complication became more and more apparent, increased precautions were taken to avoid the improper medication of our patients.

In this variety of the epidemic conditions of different years and localities, may, I think, be found a solution to the contrarieties in practice, and the discrepancies of the reports of different practitioners as to their success with any given plan of treatment.

I think it may be safely assumed, that the two great remedies relied upon at the present time in the treatment of cholera, are calomel and opium. All other remedies employed are accessories, used to meet some exigency of the case, or to fulfill some individual indications.

I think it may also be assumed, that as to the employment of these medicines, the dose, and preëminence of each, the profession are about equally divided. One division give opium freely; have learned, from experience, to give heroic doses; and they are satisfied with their success; their patients recover.

The others find calomel their sheet anchor. They give it freely, heroically, either in large doses, or in small doses so frequently repeated that the aggregate reaches the sum of the maximum doses. They are afraid of opium, morphia, and all the other compounds. Their patients bear them ill; they have soon learned that the brain is easily affected by them, and they employ them in small doses only.

In the variety of the epidemic constitution of the disease may, I believe, be found a satisfactory explanation of these vagaries of practice. It is not difficult to understand how with one physician, with one locality, and one class of patients, opium in the largest doses may be most successfully employed. While with another in a locality removed, though it be but by the space of a few streets, there may exist, from some unknown cause, such a tendency to congestion as to make him exercise the greatest caution in the administration of the same remedy.

This epidemic difference I do not believe exists merely in fancy; but am of the opinion that if the testimony of competent observers was taken, it would be found that in all the visitations of the disease with which our country has been afflicted, there have been observed differences in its character in one city as compared with another; and that if these differences were analyzed, there would be found abundant reason for the different success of similar plans of treatment.

Similar causes producing these changes in different cities, may exist to produce in a less marked degree a variety in the mode of seizure in the same city. In a disease which ravages one part of a town and exempts another, there are not wanting endemic causes in each street it visits, to produce very manifest changes in the character of the disease.

As philosophers, it becomes us rather to inquire whether such causes of difference exist, than to attack the practice which experience may have sanctioned for the time and place, and to so perfect ourselves, if possible, in diagnosis, that we may be able to arrive at intelligible, satisfactory conclusions as to the precise nature of the case before us, its probable termination and complication, in order that we may neither inflict injury by administering, or retard recovery by withholding remedies.

Before concluding this article, I wish to say a few words upon the therapeutical effects of cold, by the means of ice or water, applied to the head in cholera, and to urge it upon the profession as a remedy not undeserving trial. Its application will undoubtedly be noticed as among the remedial measures used in the cases of congestion of the brain which I have just related.

From employing it in cases of brain complications only, I gradually extended the sphere of its application, until it embraced cases of every stage, from the first seizure to that of complete collapse. And from the observation of its effects in a large number of cases, I have no hesitation in saying that it is among the most valuable means we possess of controlling the vomiting of cholera. Its influence, in some cases, was almost magical, quieting the action of the stomach, and enabling the patient to take and to retain medicines and drinks.

I append the notes of two cases, where the termination was finally fatal, where the influence exerted by this means was of the most striking character. These cases are presented alone, because there was an absence of coma, or any approach thereto.

Case XI.—Sept. 6th. A man, a Hollander, was taken to the Hospital a little before noon, in the most hopeless state of collapse. Nothing is known about the previous history of this case. He was found in a wagon in the street, by one of the Health Commssioners, having been brought by his companion from a distant part of the city in search of some public institution. He had been exposed to an intensely hot sun, but for how long a period could not be ascertained.

His condition, when received into the Hospital, was most forlorn. The vomiting and diarrhœa were so profuse and continuous, as to almost preclude his being undressed. He was covered with a cold clammy sweat.

He was got into bed as speedily as possible. There were administered to him fifty drops of laudanum; his abdomen was covered with sinapisms, and his head shaved and ice applied.

I saw him about three hours afterward, when reäction was completely established. His vomiting and diarrhœa had been *instantaneously* checked. There had been no evacuations, either way, since applying the ice. His pulse was full, skin warm, and bedewed with a warm perspiration.

His cheeks are of a deep red color, visible beneath the tan and freckles with which his face is covered. His eyes are deeply injected.

7th. Continues very comfortable; skin warm; pulse well developed and 120. Has had no vomiting. His bowels have moved off several times during the night. Is awake and completely conscious. Tongue moist and covered with a thin white coat.

Prescribed quinine and tannin, astringent injections, beef essence, and brandy.

8th. Is very comfortable. Skin and pulse the same as yesterday. Is perfectly conscious. Has no vomiting, but diarrhœa yet continues. The red cheek still remains, and the eyes continue injected.

Medication of yesterday to be continued.

9th. No vomiting, but has some diarrhœa. Pulse 120. Skin warmer than usual, producing a sharp sensation to the fingers. No tendency to coma. Is taking quinine, ammonia, and brandy.

11th. Is yet extremely prostrated, a condition increased by a profuse diarrhœa. The discharges present very much the appearance of the yolk of an egg beaten up in water. The pulse continues 120. His stomach retains nourishment and medicines.

The medication is tonics, stimulants, and astringents.

A change of weather, from extreme heat to a damp, chilly atmosphere, which took place on the 8th, has been far from beneficial to any of my patients.

12th. Not improved since yesterday. His skin is bedewed by a heavy perspiration; skin moderately warm.

He died at 6 o'clock the same evening.

An *autopsy* revealed a vast amount of disease of the intestines. The evidences of a high inflammatory condition of the large intestines were revealed, the lesions bearing a close resemblance to those manifested by several fatal cases of dysentery which happened about the same time in the house.

CASE XII.—Sept. 9th, 10½ A. M. Magnus Peiter, aged 30 years, a German emigrant just arrived in this country, and in this city only since the 4th inst., was attacked during the night. He had had no previous diarrhœa, and was brought to the Hospital about 8 o'clock this morning. He was vomiting, had diarrhœa and cramps, with loss of voice, and a scarcely distinguishable pulse—in one word, was completely collapsed. His face was flushed, but the eyes were but little injected.

Calomel, grs. xv., morphine, gr. ss., was administered, and his head shaved and ice water applied. The vomiting and diarrhœa *immediately* ceased.

5, P. M. For the last two or three hours he seems not so well. Has had cramps, and a return of the vomiting, except while he keeps the cold applied to his head. Its continual application is found to be attended with much difficulty, as he throws it off, and is in all other matters found difficult of control.

I have directed him to have

℞ Calomel, grs. x.
Morphine, gr. ss.
Quinine, grs. ij.

Ft. pulv. i. Given dry upon the tongue.

To persevere with the cold application, and administer S. quinine, grs. ij., every three hours, and brandy and beef essence *ad libitum.*

10th, 2½, P. M. His vomiting has ceased entirely since last night, and has very little, if any, diarrhœa. His skin is warm and dry; pulse 118; intelligence perfect; and he is very quiet. His prostration is, however, extreme.

Is taking stimulants and tonics.

11th. Died about 7 o'clock this morning. His diarrhœa returned with renewed force during the night. Morphia, one grain, was administered, and an injection with a drachm of laudanum, employed.

The susceptibility of this condition of the disease to the influence of opiates, was very speedily manifested in the drowsy condition of the patient, and the strong retraction of the head. These symptoms did not, however, in this case, long remain, but the medicines exerted no control over the disease.

I am aware that with many the final fatal termination of these cases will invalidate its claims for utility, and to the simultaneous application of other remedies will be ascribed a large share, if not the whole, of the influence obtained over the disease.

But these effects were manifested in a greater or less degree, in too many instances, to admit of doubt in the matter. It requires the observation of a large number of cases to enable one to judge of the influence exerted by any one particular remedial measure, and when, as I have in this instance, been able to draw the contrast between the control obtained over the disease by this single and simple means, and the power exerted over it by the other remedies previous to the addition of the cold applications, I feel a confidence in asserting for this measure, a strong claim to a prominent place among the most valuable means of combating this disease.

Its influence is to sooth the excited condition of the brain, and through it of the entire nervous system; and, consequently, control the perverted nervous action, upon which, I believe, the vomiting and diarrhœa depend.

Such was its effect in a large number of instances, several of which I have given in the course of this article. In several cases of persons fatally attacked and brought into the Hospital in a dying condition, with their skin covered with a cold, clammy sweat, its influence was manifested by its rapidly drying up the skin and diffusing a perceptible warmth over the surface. Life was apparently prolonged many hours, in a number of these instances.

Without claiming for it any power to carry every case to a successful termination, or that it is to be used to the exclusion of other remedies, I cannot but regard a measure capable of controlling vomiting, and holding the stomach in a state of absolute rest, though it be but for a few hours, enabling us to administer other remedies, and the patient to retain them, as entitled to no inconsiderable degree of merit.

Unfortunately my patients were not of a class from whom I could often obtain intelligent information of any sensations experienced in their persons, and was consequently compelled to judge from the evidence afforded by the amelioration of symptoms, of the influence exerted over them. I was, however, able in two or three instances at least, to obtain the unequivocal expressions of the patients, of the agreeable, soothing effects of the cold applications.

I have spoken of congestion of the brain, as *a stage* of cholera, and as dependent upon irritation of the brain excited in the *course* of the disease. I am now prepared to advance a step farther, and claim, that the first manifestations of the specific cause of cholera, upon the human organism, be that cause what it may, is upon the brain, and through it upon the nervous centers. That the morbific influence of the blood poison is first upon the sensorium, and that the irritation there and thus excited, is manifested in the perverted action of the organs, inducing diarrhœa and vomiting, which are the effects, and not the cause of the disease. And that some peculiarity of the epidemic seizure governs the amount of the intensity of the shock upon the brain, determining the extent of lesions there produced, and the degree of consciousness of the patient.

If this view be correct, we can understand how the application of cold may be made to assume a prominent rank among our remedies. By soothing and quieting down the excited condition of the brain, controlling and relieving congestion, we enable the great nervous center to reäct and cast off the morbific influences exerted upon it, and conduce to perfect restoration.

To obtain these beneficial results, its application in the earliest stages of the disease is indicated before effusion has taken place, or lesion of structure or function has occurred. With such application, and being taught to look for the flushed face and injected eye, and to regard them when present with suspicion, may we not hope to control this fearful disease, and to stave off its no less fearful complication, Congestion of the Brain?

